# Threshold Effects of Straw Returning Amounts on Bacterial Colonization in Black Soil

**DOI:** 10.3390/microorganisms13081797

**Published:** 2025-07-31

**Authors:** Genzhu Wang, Wei Qin, Zhe Yin, Ziyuan Zhou, Jian Jiao, Xiaohong Xu, Yu Zhang, Xing Han

**Affiliations:** 1 Institute of Sediment Research, China Institute of Water Resources and Hydropower Research, Beijing 100038, China; wanggz@iwhr.com (G.W.); yinzhe-2002@163.com (Z.Y.); jiaojian@iwhr.com (J.J.); 2 State Key Laboratory of Water Cycle and Water Security, China Institute of Water Resources and Hydropower Research, Beijing 100038, China; 3 Institute of Desertification Studies, Chinese Academy of Forestry, Beijing 100091, China; zhouziyuan@caf.ac.cn; 4 Jilin Provincial Bureau of Soil and Water Conservation, Changchun 130028, China; kexueyanjiu666@126.com; 5 Institute of Soil and Water Conservation of Jilin Province, Changchun 130033, China; zy0431@163.com; 6 College of Resources and Environment, Jilin Agricultural University, Changchun 130118, China; xingh@jlau.edu.cn

**Keywords:** black soil of Northeast China, corn, soil bacterial community, straw returning amounts

## Abstract

Straw returning (ST) significantly improves soil quality and profoundly impacts soil microorganisms. However, the effects of different ST application amounts on the soil bacterial community remain unclear, and more studies on optimal ST application amounts are warranted. This study aimed to investigate the bacterial diversity and composition, as well as physicochemical properties, of soil in a corn field with 5-year ST amounts of 0, 3, 4.5, 5, and 6 t/hm^2^, respectively. The results indicated that ST significantly reduced soil bulk density and increased soil pH and nutrients. Meanwhile, ST had a significant effect on the bacterial composition, and the bacterial diversity increased significantly after ST. The relative abundance of *Proteobacteria* and *Acidobacteria* increased dramatically, whereas that of *Actinobacteria* significantly decreased after ST. The amount of ST had threshold effects on soil physicochemical properties and the dominant bacterial phyla. Moreover, the co-occurrence networks indicated that bacterial stability first increased and then decreased with the increase in ST amounts. Soil organic carbon and total nitrogen concentrations were the main drivers of bacterial diversity, whereas soil pH and total nitrogen concentrations were the main drivers of bacterial composition. This study strengthens the fact that ST amounts have threshold effects on the soil physicochemical properties and soil microorganisms, and ST amounts of 3–5 t/hm^2^ were appropriate.

## 1. Introduction

Food security is crucial for both economic development and social stability [1]. The black soil area in Northeast China, one of the major black soil distribution regions in the world, serves as a key commodity grain production base and plays a significant role in China’s food security [2,3]. Recently, soil erosion and reduced fertility in black soil areas have become severe due to artificial, unreasonable land use and natural factors [4,5,6]. Straw returning (ST) is a typical protective tillage measure that can control soil erosion, improve soil fertility and soil structure, and also solve the environmental problems caused by direct burning [4,7]. The role of ST technology in improving soil quality and increasing crop yields has received widespread attention in recent years, driven by the growing demand for sustainable agricultural development [3,8].

Previous studies have demonstrated that ST can improve soil structure and nutrient concentrations, which also indirectly enhance the microbial community structure [9,10]. However, the slow decomposition rate of straw poses a significant challenge to the large-scale adoption of ST in fields, as it can reduce soil fertility and threaten both national food and ecological security [11,12]. The long-term return of large amounts of straw to the field may lead to soil health issues, including increased soil acidification, nutrient imbalance (increased nitrogen fixation and decreased available nutrients), and the enrichment of pathogenic microorganisms [4,13,14]. However, the optimal ST application rates for improving soil quality and enhancing grain production remain unclear [4].

Soil microorganisms are essential indicators for evaluating soil health and play a crucial role in soil nutrient cycling and straw decomposition [15,16]. Meanwhile, soil microbial communities are susceptible to changes in their living environment, and soil pH and nutrients may be crucial drivers of microbial colonization [17,18]. Additionally, microbial adaptability to ST may also be influenced by enzyme activity and functional microbial traits [19,20,21]. Notably, soil bacterial communities are essential for their contribution to soil biodiversity [22], and drive vital ecosystem functions, such as nutrient cycling, carbon sequestration, and degradation [23,24,25]. Moreover, compared to fungi, bacteria are more susceptible to environmental changes and have received more attention [26,27]. Previous studies indicate that bacterial diversity can increase [28,29], decrease [30,31,32], or remain unchanged with ST [33]. In addition, the bacterial composition responds differently to ST [34,35,36]. However, the patterns and drivers of soil bacterial communities under different ST amounts remain unclear [11,29]. Thus, further studies are warranted to investigate the effects of different ST amounts on soil bacterial colonization [12].

This study examined the composition and diversity of bacterial communities in corn fields with different ST amounts in Northeastern China. Additionally, the corresponding soil physical and chemical properties were simultaneously assessed. The aims of this study were as follows: (1) to elucidate the patterns of bacterial diversity and composition in corn fields with different ST application amounts, (2) to explore the effects of environmental factors on bacterial composition and diversity, and (3) to test the differences in the bacterial network topological features with different ST application amounts.

## 2. Methods

### 2.1. Site Description

The study sites were located in Xingmu, which is a small watershed in Dongliao County (125°35′–125°50′ E, 43°13′–43°36′ N), Jilin Province. The drainage basin is situated in the upper reaches of the East Liaohe River, at the margin of Paektu Mountain, which is characterized by typical low mountain and hilly landforms in the northeast black soil region. The study areas have a continental monsoon climate of cold temperate zones. The average annual precipitation is 658.1 mm, and the average annual temperature is 5.2 °C. The highest temperature is 38 °C, and the lowest temperature is −40 °C. The vegetation type belonged to the Changbai Mountain flora, comprising natural secondary forests, artificial forests, herbs, and crops. According to the Food and Agriculture Organization classification system, the soil type in the study area was classified as black soil.

### 2.2. Experimental Design and Soil Sampling

Conservation tillage experiments have been conducted at the Xingmu Soil and Water Conservation Experimental Base since 2011. The crops selected in this study were corn with different ST application amounts. The total ST amount selected for this study was 6 t/hm^2^ because it is the average and most commonly used ST amount in Dongliao County. Five different ST application amounts, which were 0 t/hm^2^ (0%), 3 t/hm^2^ (50%), 4.5 t/hm^2^ (75%), 5 t/hm^2^ (83%), and 6 t/hm^2^ (100%), were set in the straw mulching area to explore the appropriate ST amount. Three replicates were set for each treatment. The soil samples were collected in 2016, indicating that the ST study had been conducted for 5 years and was sufficient to test the effects on soil physicochemical properties and the soil microbiome [3,12]. Nine soil samples were randomly collected using an “S”-shaped method and blended to yield a single composite soil specimen. A total of 15 soil samples (5 ST amounts × 3 replicates) were collected. During the sampling, the topsoil was first removed, and samples were then collected from the plow layer within a root circumference of 5–20 cm. The soil bulk density (BD) was measured using a ring knife and an aluminum box. Once the mixed samples were cleared of small stones, roots, and other debris, each soil sample was divided into two parts. Some of these were air-dried and sieved through a 2 mm sieve for subsequent chemical index measurement, whereas the remaining were frozen and stored in a refrigerator at −80 °C for DNA extraction.

### 2.3. Analysis of Soil Chemical Index and Bacterial DNA

Potentiometry (water-to-soil ratio of 2.5:1) and a pH meter were used to measure the soil pH [37]. The external heating method of potassium dichromate was used to measure the soil organic carbon (SOC) content [37]. The contents of soil-available phosphorus (AP), total nitrogen (TN), and total phosphorus (TP) were determined using an automatic chemical analyzer. The available nitrogen (AN) was extracted with a 1:10 ratio of soil and 2 mol of KCl solution with 1 h of shaking before being measured by the Kjeldahl method. The soil-available potassium (AK) content was determined using the flame photometer method. The soil total potassium (TK) content was determined using ICP-MS (EDX4500P), which was produced by Jiangsu Skyray Instrument Company Limited in Kunshan, Jiangsu, China. The methods for soil DNA extraction, polymerase chain reaction amplification, and data processing are shown in Attachment S1 (Supplementary Materials).

### 2.4. Statistical Analyses

The least significant difference and one-way analysis of variance multiple comparison tests were conducted to assess soil properties, bacterial diversity, and the dominant phyla with a relative abundance >1.0% (*p* < 0.05). The Shapiro–Wilk test was conducted to determine the normality of data distribution. Bray–Curtis dissimilarity-based principal coordinates analysis (PCoA) was conducted to assess the soil bacterial composition [38]. PERMANOVA analysis was used to explore the effects of ST amounts on the composition of soil bacteria [38]. Linear regression analysis was utilized to explore the associations between bacterial diversity and environmental factors. Boosted regression tree (BRT) was used to assess the relative effects of environmental factors on the diversity and composition of soil bacteria [39,40]. Bacterial diversity was assessed using the Shannon–Wiener index [41,42], and bacterial community composition was represented by PCoA1. While using the BRT models, only the environmental factors that significantly influenced the bacterial diversity and composition were selected. Redundancy analysis was conducted to quantify the effects of environmental factors on bacterial-dominant phyla [42]. The co-occurrence network was applied to perform the variability analysis of the co-occurrence network in the corn fields with different ST amounts for the selected target species. The Spearman correlation analysis was performed, and data with correlation coefficients greater than 0.6 and *p* values less than 0.05 were selected. The Gephi version 0.92 software (https://gephi.org/, accessed on 15 May 2024) was used to visualize and analyze co-occurrence network results [43].

## 3. Results

### 3.1. The Effects of ST Treatment on the Physicochemical Characteristics of Soil

**Figure 1:**
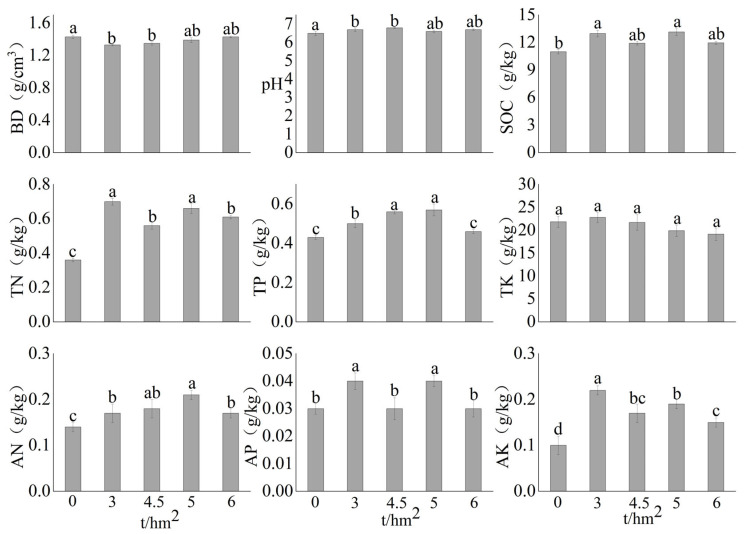
Soil physical and chemical properties of the corn field after straw returning. BD: bulk density; SOC, TN, TP, TK, AN, AP, and AK represent soil organic carbon, total nitrogen, total phosphorus, total potassium, available nitrogen, available phosphorus, and available potassium, respectively. Lowercase letters indicate significant differences among different ST amounts (*p* < 0.05).

Small amounts (3 and 4.5 t/hm^2^) of ST significantly reduced the soil bulk density. In contrast, larger amounts (5 and 6 t/hm^2^) caused no significant changes (Figure 1). The soil pH and SOC, TP, and AP contents significantly increased after treating the field with a small amount of ST, whereas no significant changes were noted after treatment with a large amount of ST. The soil TN, AN, and AK contents significantly increased after ST, and the increase significantly decreased after treatment with a large amount of ST. The change in soil TK content after treating the field with ST was not significant.

### 3.2. The Effects of ST Treatment on Soil Bacterial Diversity

**Figure 2:**
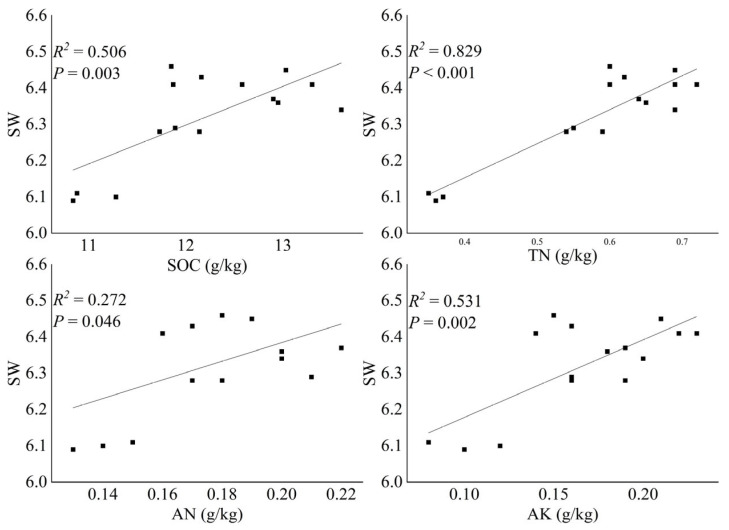
Bivariate relationships between the bacterial alpha diversity and environmental factors (*n* = 15). Only the environmental factors significantly influencing bacterial diversity are shown (*p* < 0.05). SW, SOC, AN, and AK represent the Shannon–Wiener index of soil bacteria, soil organic carbon, available nitrogen, and available potassium, respectively.

**Figure 3:**
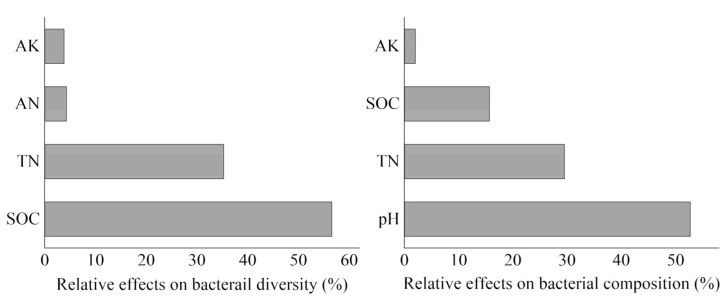
The relative contributions of different environmental factors to the composition and diversity of the soil bacterial community. SOC, TN, AN, and AK represent soil organic carbon, total nitrogen, available nitrogen, and available potassium, respectively.

After ST treatment, the Chao1, Shannon–Wiener index, OTU number, and ACE all significantly increased (Table 1). The Chao1, Shannon–Wiener index, and ACE first decreased and then increased with the increase in ST amounts. No significant difference was found in the OTU numbers in corn fields after treatment with different ST amounts. Simple linear regressions indicated that bacterial diversity increased significantly with the increase in SOC (*R*^2^ = 0.506, *p* = 0.003), soil TN (*R*^2^ = 0.829, *p* < 0.001), AN (*R*^2^ = 0.272, *p* = 0.046), and AK (*R*^2^ = 0.531, *p* = 0.002) contents (Figure 2). The BRT model indicated that the bacterial diversity was mostly affected by SOC (56.6%), followed by TN (35.3%), AN (4.3%), and AK (3.8%) (Figure 3).

### 3.3. The Effects of ST Treatment on Soil Bacterial Composition

**Figure 4:**
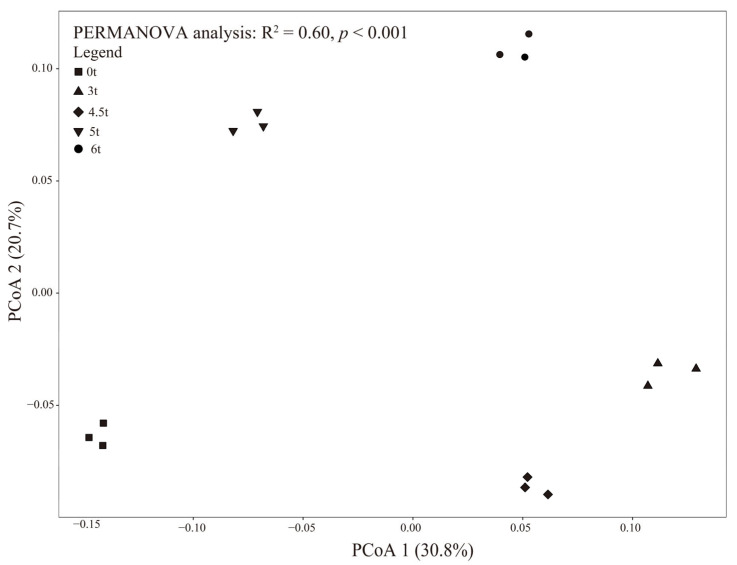
Principal coordinates analysis of soil bacterial composition based on the Bray–Curtis distance values. The impact of different ST amounts on the bacterial composition was assessed via a permutational multivariate analysis of variance.

**Figure 5:**
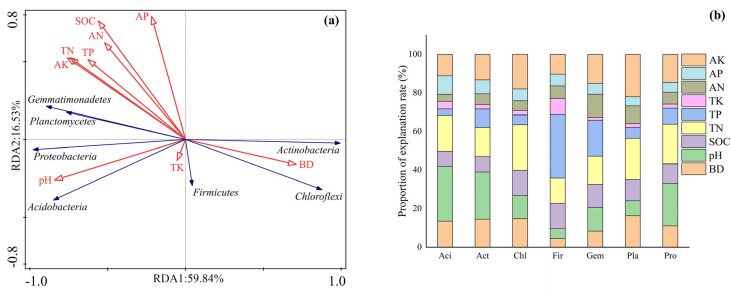
Ordination plots were conducted for the redundancy analysis to quantify the effects of environmental factors (red arrows) on the dominant phyla (blue arrows). BD represents soil bulk density; SOC, TN, AN, TP, AP, TK, and AK represent soil organic carbon, total nitrogen, available nitrogen, total phosphorus, available phosphorus, total potassium, and available potassium, respectively.

The PCoA and PERMANOVA analyses showed that different ST application amounts significantly influenced the soil bacterial composition (*p* < 0.05) (Figure 4). The main phyla were *Proteobacteria* (24.82–31.59%), *Actinobacteria* (25.3–35.6%), *Acidobacteria* (8.13–10.59%), *Chloroflexi* (8.13–11.4%), *Gemmatimonadetes* (6.17–8.14%), *Firmicutes* (2.27–2.28%), and *Planctomycetes* (4.78–6.29%) (Table 2). The relative abundance of *Proteobacteria*, *Acidobacteria*, *Gemmatimonadetes*, and *Planctomycetes* significantly increased after ST treatment, whereas those of *Actinobacteria* and *Chloroflexi* displayed the opposite trend. The bacterial composition was affected mostly by pH (52.7%), followed by TN (29.5%), SOC (15.7%), and AK (2.1%) contents (Figure 3). The soil bulk density, pH, and SOC, TN, and AK contents significantly affected the dominant phyla (Figure 5).

### 3.4. Effects of ST on Soil Bacterial Co-Occurrence Network

**Figure 6:**
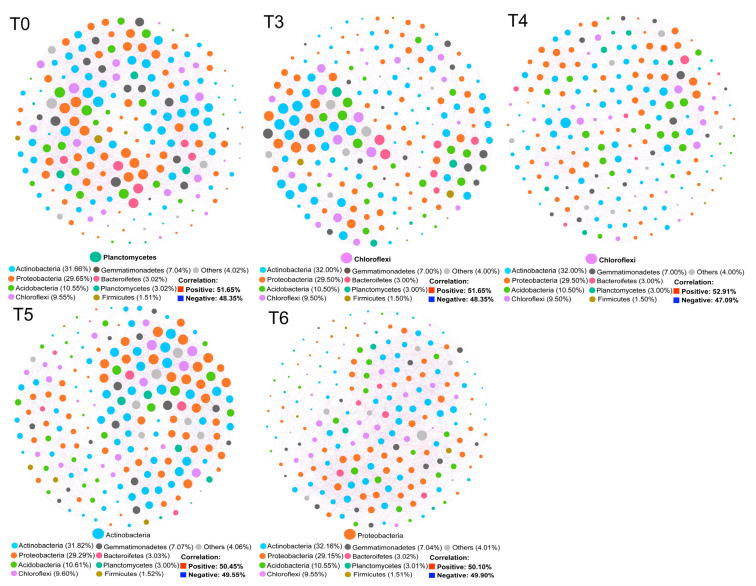
Network analysis of soil bacterial communities varying with different ST amounts. The red links indicate a positive correlation (red squares represent the percentage of positively correlated line counts in the common network), and the blue links indicate a negative correlation (blue squares represent the percentage of negatively correlated line counts in the common network). The larger the circle for a species, the more connections that circle has and the more it is related to other species, highlighting the core species in the community correlation. Colored nodes represent core phyla with >1% relative abundance, and gray nodes indicate phyla with <1% relative abundance. T0, T3, T4, T5, and T6 represent corn fields with ST amounts of 0, 3, 4.5, 5, and 6 t/hm^2^, respectively.

**Figure 7:**
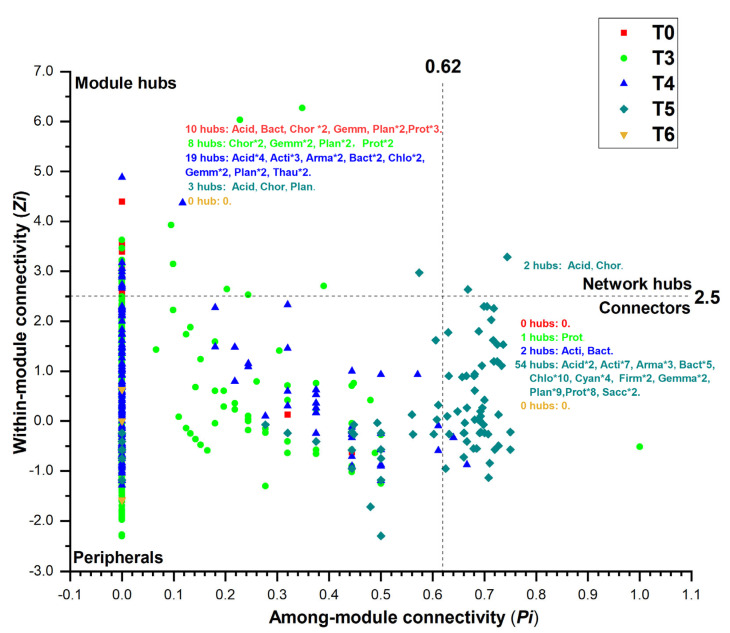
Topological node property analysis of soil bacterial co-occurrence networks under different ST amounts. *Zi* and *Pi* thresholds were set at 2.5 and 0.62, respectively. T0, T3, T4, T5, and T6 represent ST application rates of 0, 3, 4.5, 5, and 6 t/hm^2^, respectively, in corn fields. Acid, Acidobacteria; Acti, Actinobacteria; Arma, Armatimonadetes; Chlo, Chloroflexi; Gemm, Gemmatimonadetes; Plan, Planctomycetes; Prot, Proteobacteria; Thau, Thaumarchaeota.

The co-occurrence network of the bacterial community was assessed at the OTU level. *Proteobacteria*, *Actinobacteriota*, *Chloroflexi*, and *Planctomycetota* were the core phyla (Figure 6). Specifically, *Planctomycetota*, *Actinobacteriota*, and *Proteobacteria* were the core phyla in soils treated with ST amounts of 0, 5, and 6 t/hm^2^, respectively. *Chloroflexi* was the core phylum of soils treated with ST amounts of 3 and 4.5 t/hm^2^. Under a constant similarity threshold, total nodes, total links, positive links, connectedness, harmonic geodesic distance, and average path distance first increased and then decreased with the increase in ST amounts (Table 3). The high modularity values (0.856–0.938) across treatments indicated that the bacterial symbiotic networks in corn fields were highly modular. According to the *Zi* and *Pi* values from the network topology analysis, two network hubs were found under the ST 5 t/hm^2^ treatment, suggesting the highest connectivity in the entire network (Figure 7). The number of module hubs in corn fields treated with ST amounts of 0, 3, 4.5, 5, and 6 t/hm^2^ was 10, 8, 19, 3, and 0, respectively. The corresponding number of connectors in the networks was 0, 1, 2, 54, and 0. Most bacterial nodes were affiliated with *Proteobacteria*, *Actinobacteria*, *Acidobacteria*, and *Chloroflexi*.

## 4. Discussion

### 4.1. Effects of Different ST Application Amounts on Soil Physicochemical Properties

The results of this study demonstrate that treatment with ST improved the soil’s physical structure and effectively reduced the soil bulk density, which is consistent with previous findings [44]. A small amount of ST (3–4.5 t/hm^2^) significantly improved the soil structure by reducing the bulk density, likely due to enhanced organic matter input and microbial activity that promoted soil aggregation [35]. However, higher ST amounts (5–6 t/hm^2^) did not further decrease the bulk density, suggesting a saturation point where additional organic inputs no longer improved soil porosity. The results of this study also indicated that ST significantly increased the pH and SOC and improved the nitrogen, phosphorus, and potassium contents of soil, which is consistent with previous findings [4,9,10]. In addition, soil pH and nutrients (e.g., SOC, TN, TP, AN, AP, and AK) decreased with the increase in ST treatment amounts. These outcomes resulting from different ST application amounts might be explained by three possible factors. First, incorporating moderate straw into soil initially buffered acidity by releasing alkaline cations (e.g., K^+^ and Ca^2+^) during decomposition [35]. However, higher ST rates increased organic acid production and CO_2_ release due to the prolonged microbial decomposition of straw, exacerbating soil acidification [8,11]. Second, the initial rise in SOC was attributed to humification and the formation of a stable carbon pool [4]. However, excessive straw might accelerate microbial mineralization, leading to carbon loss through respiration. Similarly, TN and TP contents initially increased due to the retention of straw-derived nutrients but declined at higher ST application rates, possibly due to microbial immobilization (particularly of N) and P fixation by Al/Fe oxides under acidic conditions [4,7]. In addition, moderate straw input improved nutrient availability by enhancing microbial turnover (e.g., N mineralization and P solubilization) [4,11]. However, excessive straw probably induced nutrient imbalances, such as N immobilization due to a high carbon/nitrogen (C:N) ratio of straw and K^+^ leaching resulting from cation displacement. In summary, the results of this study indicate an apparent threshold effect of ST amounts on the physicochemical properties of black soil in Northeast China.

### 4.2. Effects of Different ST Application Amounts on Soil Bacterial Diversity

Significant increases in the Chao1, Shannon–Wiener index, OTU number, and ACE index following ST treatment (Table 1) indicated that organic amendments generally enhanced microbial diversity, which is consistent with previous findings in agricultural ecosystems [28,29]. The observation that OTU numbers remained stable across different ST application rates, whereas other diversity indices fluctuated, suggests that ST treatment primarily affects the relative abundance rather than the total number of bacterial taxa. This pattern may reflect the ecological succession processes where initial carbon inputs favor fast-growing r-strategists (e.g., *Proteobacteria*), temporarily reducing diversity metrics until K-strategists (e.g., *Acidobacteria*) become established [45]. In this study, the linear regression analyses identified SOC, TN, AN, and AK as key drivers of bacterial diversity (Figure 2), with the BRT model further quantifying their relative importance (Figure 3). The dominant influence of SOC aligns with the energy-diversity hypothesis, which posits that increased carbon availability promotes greater microbial niche differentiation [42]. SOC is the main energy source for soil bacteria, which obtains the energy needed for growth, reproduction, and the maintenance of life activities by decomposing SOC [42,46]. In addition, TN, AN, and AK were all crucial nutrients for the growth and survival of bacterial communities [42,46,47]. Nitrogen is a key component of bacterial cell structures such as proteins and nucleic acids, and potassium is an activator of many bacterial enzymes and participates in bacterial metabolic processes [19,28,31]. The roles of TN, AN, and AK highlight the critical balance between carbon and nutrient availability in shaping microbial communities, as high C:N ratios may lead to nutrient immobilization and competitive exclusion [45,48].

### 4.3. Effects of Different ST Application Amounts on Soil Bacterial Composition

The PCoA and PERMANOVA results clearly demonstrated that ST significantly restructured soil bacterial communities in Northeast China’s black soil (Figure 4, *p* < 0.05). The soil pH was the predominant driver of the bacterial composition, which was similar to previous findings [42,49,50]. Soil pH can not only directly affect bacterial colonization through the physiological constraints on bacterial taxa but also can affect bacterial colonization indirectly via soil properties, such as salinity, metal solubility, and nutrient content and availability [42,49,50]. *Proteobacteria*, *Actinobacteria*, and *Acidobacteria* were the most important bacterial phyla, which is consistent with previous results [51,52]. *Actinobacteria* and *Proteobacteria* were regarded as fast-growing copiotrophs and *Acidobacteria* were considered oligotrophic bacteria [53,54]. The increased relative abundance of *Proteobacteria* following ST aligns with their known copiotrophic characteristics, as this phylum contains many fast-growing, carbon-responsive taxa capable of rapidly utilizing fresh organic inputs [42,45]. Similarly, the enrichment of Acidobacteria (8.13–10.59%) suggests that these typically oligotrophic organisms might benefit from the gradual decomposition of more recalcitrant straw components [42,45]. The contrasting decline in *Actinobacteria* (25.3–35.6%) and *Chloroflexi* (8.13–11.4%) abundance may reflect niche displacement, as these slow-growing phyla tend to dominate in low-carbon environments [45]. The reduced abundance of *Actinobacteria*, despite their known capacity to degrade complex plant polymers, indicates competitive suppression under conditions of high carbon availability combined with altered pH and nutrient status [42]. The finding that pH, TN, SOC, and AK were the primary drivers of community composition (Figure 3) supports the fact that ST mediated bacterial assembly through both direct (carbon input) and indirect (soil property modification) pathways. The soil pH, TN, SOC, and AK jointly shaped the composition of soil bacterial communities through a complex interaction network. The pH value, as the most direct environmental factor, had a wide range of impacts on the physiological and chemical processes of bacteria [45,50]. The strong influence of pH likely reflects its fundamental role in shaping microbial niche spaces, as demonstrated by the increased abundance of *Proteobacteria* and *Acidobacteria* under elevated pH conditions [49,50]. TN and SOC, as important nutrients for bacterial growth, directly affected the biomass and metabolic activity of bacteria [31,42,48]. The impact of AK was relatively weak, likely because it mainly affected bacterial communities through indirect pathways, such as maintaining enzyme activity [21,50,52].

### 4.4. Effects of Different ST Amounts on Soil Bacterial Co-Occurrence Network

Co-occurrence network results reflected the indirect and direct competition or cooperation relationships among bacterial taxa [55,56,57,58]. Higher connective numbers among coupled bacterial taxa indicated a greater degree of network cohesion [59,60]. The number of edges, nodes, betweenness, and average degree first increased and then decreased with increasing ST amounts, illustrating the threshold effects of ST amounts on the coupling within the functional bacterial taxa. The negative and positive correlations in the co-occurrence network suggest the presence of competitive and cooperative relationships between pairwise coupling bacterial taxa, respectively [61,62]. Consistent with previous results, a positive correlation was dominant in our co-occurrence network, indicating that cooperation dominated among the bacterial taxa in the corn fields with different ST amounts [48,63]. The dominant phyla (e.g., *Proteobacteria*, *Actinobacteria*, *Acidobacteria*, and *Chloroflexi*) were closely associated with other bacterial taxa, playing crucial roles in the bacterial networks and exerting strong influences on bacterial communities. *Proteobacteria* and *Actinobacteria* are considered copiotrophic bacteria that can increase soil fertility and crop growth [53,64,65]. *Acidobacteria* can degrade organic matter and survive and grow well in carbon-deficient conditions [66,67]. Bacterial networks with higher structural complexity are more capable of withstanding environmental stresses [68]. The bacterial network indicated enhanced niche differentiation and a more complex topological structure after treatment with moderate ST amount, which might have resulted from the increase in SOC content and soil fertility in farmlands [65,69]. However, the SOC and soil fertility decreased with excessive ST amounts, leading to intensified competition among microorganisms and the decreased stability of microbial communities [45,68]. Thus, the results indicate that the bacterial stability first increased and then decreased with the increase in ST amounts, suggesting threshold effects of ST amounts on soil bacterial stability.

## 5. Conclusions

This study demonstrates that ST amounts exhibit threshold effects on both soil physicochemical properties and the soil microbiome in black soil corn fields. Although ST significantly decreased soil BD and improved soil pH and nutrient concentrations, these benefits were not linearly proportional to the ST amounts. Specifically, the findings revealed that ST amounts of 3–5 t/hm^2^ provided the most optimal balance, promoting soil health and microbial diversity while mitigating the potential detrimental effects associated with excessive straw application. This range fostered beneficial shifts in bacterial community composition, increasing the relative abundance of *Proteobacteria* and *Acidobacteria* while decreasing the abundance of *Actinobacteria*. The observed changes in soil bacterial stability, which initially increased and subsequently decreased with the increase in ST amounts, highlight the need for careful management of ST application. Furthermore, SOC and TN were identified as the key drivers of bacterial diversity, whereas soil pH and TN primarily influenced bacterial composition. These findings underscore the importance of optimizing ST application rates to maximize soil health and support sustainable agricultural practices in black soil regions. It is recommended that farmers in this region should target ST amounts of 3–5 t/hm^2^ to achieve optimal soil health and microbial balance.

## Figures and Tables

**Table 1:** Soil bacterial diversity features after treatment with different ST amounts (*n* = 3).

Straw Returning Amount	Chao1	Shannon	OTUs	ACE
0 t/hm^2^	2197 ± 23 c	6.1 ± 0.01 d	1715 ± 38 b	2182 ± 18 c
3 t/hm^2^	2661 ± 77 a	6.42 ± 0.02 a	2197 ± 77 a	2666 ± 80 a
4.5 t/hm^2^	2415 ± 48 b	6.28 ± 0.01 c	1944 ± 40 a	2422 ± 58 b
5 t/hm^2^	2686 ± 120 a	6.35 ± 0.02 b	2152 ± 120 a	2664 ± 106 a
6 t/hm^2^	2608 ± 111 ab	6.43 ± 0.03 a	2113 ± 166 a	2593 ± 138 ab

Values are shown as the mean ± standard error. Lowercase letters indicate significant differences among different ST amounts (*p* < 0.05).

**Table 2:** The relative abundance (%) of the dominant soil bacterial phyla (>1%) after treatment with different ST amounts.

Dominant Phyla	0 t/hm^2^	3 t/hm^2^	4.5 t/hm^2^	5 t/hm^2^	6 t/hm^2^
*Proteobacteria*	24.82 ± 0.45 d	30.88 ± 0.66 ab	31.59 ± 0.1 a	28.46 ± 0.31 c	29.7 ± 0.34 bc
*Actinobacteria*	35.6 ± 0.65 a	26.88 ± 0.42 c	25.3 ± 0.44 c	29.36 ± 0.09 b	28.87 ± 0.44 b
*Acidobacteria*	8.13 ± 0.24 b	10.47 ± 0.2 a	10.59 ± 0.34 a	8.4 ± 0.09 b	9.93 ± 0.23 a
*Chloroflexi*	11.4 ± 0.16 a	8.13 ± 0.16 c	9.13 ± 0.19 b	9.29 ± 0.07 b	9.51 ± 0.18 b
*Gemmatimonadetes*	6.17 ± 0.11 c	7.68 ± 0.2 ab	8.14 ± 0.24 a	7.65 ± 0.09 ab	7.32 ± 0.16 b
*Firmicutes*	2.69 ± 0.15 a	2.29 ± 0.17 a	2.88 ± 0.14 a	2.5 ± 0.16 a	2.27 ± 0.08 a
*Planctomycetes*	4.78 ± 0.08 b	6.29 ± 0.28 a	5.98 ± 0.19 a	5.55 ± 0.08 ab	5.69 ± 0.35 ab

Values are shown as the mean ± standard error. Different letters indicate significant differences (*p* < 0.05) among the different ST amounts.

**Table 3:** The topological features of phylogenetic molecular networks and corresponding random networks of the bacterial community after treatment with different ST amounts.

		0 t/hm^2^	3 t/hm^2^	4.5 t/hm^2^	5 t/hm^2^	6 t/hm^2^
Empirical networks	Similarity threshold	0.85	0.85	0.85	0.85	0.85
	Total nodes	543	1068	650	106	329
	Total links	805	3313	753	708	592
	*R*^2^ of power law	0.912	0.765	0.949	0.715	0.836
	Average clustering coefficient	0.179	0.197	0.082	0.342	0.61
	Connectedness	0.042	0.679	0.201	1	0.011
	Harmonic geodesic distance	2.535	6.609	5.798	1.944	1.001
	Average path distance	4.034	8.311	8.031	2.18	1.002
	Modularity	0.926	0.917	0.888	0.856	0.938
Random networks	Random average clustering coefficient	0.008 ± 0.003	0.012 ± 0.002	0.004 ± 0.002	0.452 ± 0.019	0.007 ± 0.002
	Random average path distance	4.997 ± 0.065	3.852 ± 0.017	6.173 ± 0.112	2.180 ± 0.017	4.672 ± 0.058
	Modularity	0.633 ± 0.006	0.374 ± 0.004	0.763 ± 0.006	0.150 ± 0.005	0.633 ± 0.006

## Data Availability

All data generated or analyzed during this study have been uploaded. The data of environmental factors, bacterial diversity and composition, and dominant phyla are shown in Attachment S2. The OTU data are shown in Attachment S3.

## Supplementary Materials

The following supporting information can be downloaded at https://www.mdpi.com/article/10.3390/microorganisms13081797/s1. Attachment S1: Soil DNA extraction, polymerase chain reaction amplification, and data processing; Attachment S2: The data of environmental factors, bacterial diversity and composition, and dominant phyla; Attachment S3: The OTU data.

## Abbreviations

The following abbreviations are used in this manuscript:

SOC	Soil organic carbon
BD	Bulk density
TN	Total nitrogen
TP	Total phosphorus
TK	Total potassium
AN	Available nitrogen
AP	Available phosphorus
AK	Available potassium
BRT	Boosted regression tree
PCoA	Principal coordinates analysis
