# Supplementary material for: Threshold Effects of Straw Returning Amounts on Bacterial Colonization in Black Soil
Source: Microorganisms. 2025 Jul 31;13(8):1797. doi: 10.3390/microorganisms13081797 (PMC12388704; doi:10.3390/microorganisms13081797)
Supplement: Supplementary file 1 [file microorganisms-13-01797-s001.zip › Attachment S1.pdf]

*Soil DNA extraction, polymerase chain reaction amplification, and data processing*

Each soil sample was weighed with 0.5 g of fresh soil, and the FastDNA spin kit for soil (MP Biomedicals, CA, USA) was utilized to extract soil DNA. The extracted total soil DNA was detected by 1% agarose gel electrophoresis, and the DNA concentration was determined using a NanoDrop 2000 spectrophotometer (Thermo Fisher Scientific, MA, USA). The DNA was stored at 20℃ for future use. The specific primers f515/r907 were used for polymerase chain reaction (PCR) amplification of bacterial 16S rRNA v4-v5 region. The primer sequence of each sample contained a 7-bp specific tag sequence for distinguishing different samples. The PCR amplification products were identified and separated by 1% agarose gel electrophoresis. The PCR products were purified using the agarose gel DNA purification kit (Takara). The purified products were used for subsequent sequencing. The purified genomic DNA was collected, and the library template was enriched by PCR amplification to generate single-stranded DNA fragments. DNA clusters were generated by PCR amplification, and the DNA amplicons were linearized into single strands. The surface of the reaction plate was scanned using a laser, and the nucleotide species polymerized in the first round of reaction of each template sequence were read. The fluorescence signal results collected in each round were counted, and the sequence of template DNA fragments was obtained.
